# Intracranial Pressure Monitoring—Review and Avenues for Development

**DOI:** 10.3390/s18020465

**Published:** 2018-02-05

**Authors:** Maya Harary, Rianne G. F. Dolmans, William B. Gormley

**Affiliations:** 1 Computational Neuroscience Outcomes Center, Department of Neurosurgery, Brigham and Women’s Hospital, Harvard Medical School, Boston, MA 02115, USA; mharary@partners.org (M.H.); r.g.f.dolmans@students.uu.nl (R.G.F.D.); 2 Department of Neurosurgery, University Medical Center, 3584 CS Utrecht, The Netherlands

**Keywords:** intracranial pressure monitoring, neurocritical care, cerebral compliance

## Abstract

Intracranial pressure (ICP) monitoring is a staple of neurocritical care. The most commonly used current methods of monitoring in the acute setting include fluid-based systems, implantable transducers and Doppler ultrasonography. It is well established that management of elevated ICP is critical for clinical outcomes. However, numerous studies show that current methods of ICP monitoring cannot reliably define the limit of the brain’s intrinsic compensatory capacity to manage increases in pressure, which would allow for proactive ICP management. Current work in the field hopes to address this gap by harnessing live-streaming ICP pressure-wave data and a multimodal integration with other physiologic measures. Additionally, there is continued development of non-invasive ICP monitoring methods for use in specific clinical scenarios.

## 1. Introduction

**Figure 1:**
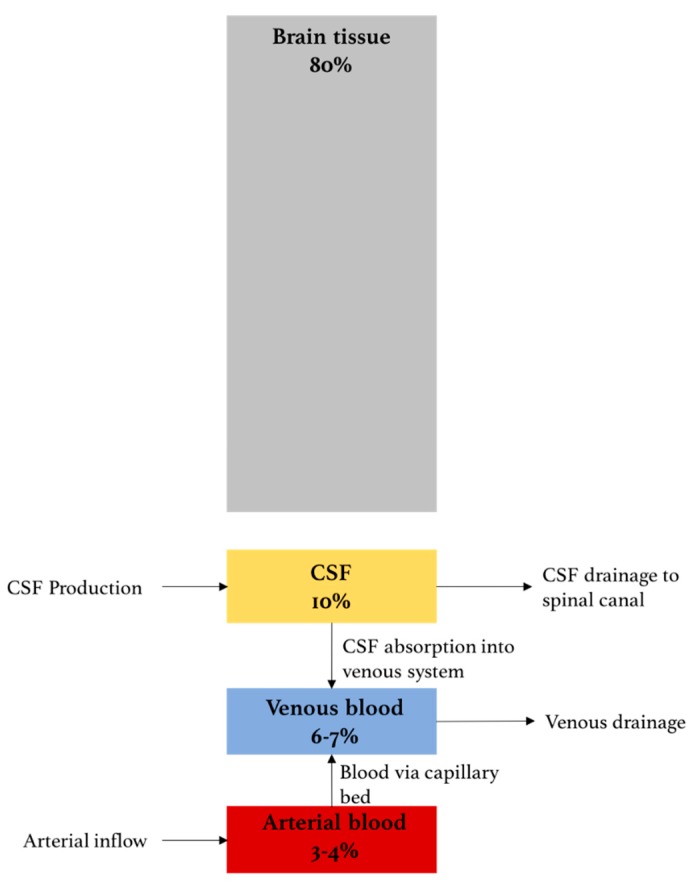
The Monro–Kellie model for the contents of the intracranial compartment. ‘Brain tissue’ includes neurons, glia, extracellular fluid and cerebral microvasculature. ‘Venous’ and ‘Arterial blood’ represents the intracranial blood volume in macro-vasculature and cerebral venous sinuses. ‘CSF’ includes ventricular and cisternal CSF.

The systematic discussion of intracranial pressure (ICP) and its determinants dates back to the work of Scottish anatomist Alexander Monro and a compatriot surgeon, George Kellie, at the turn of the 18th century. Their model for ICP, the Monro–Kellie doctrine, which was later refined by American neurosurgeon, Harvey Cushing, details the basic principles that govern ICP [1,2,3]. Principally, the volume of the intracranial cavity is constant under normal conditions, and, therefore, the maintenance of a steady ICP depends on the volume of its contents. The intracranial contents include (1) brain tissue; (2) blood; and (3) cerebrospinal fluid (CSF) (Figure 1) [4,5,6]. As brain tissue is incompressible, steady ICP requires balancing the in- and outflow of the fluid components; namely, there must be a balance between the inflow of arterial blood and the outflow of venous blood from the head, as well as between the rate of CSF production and drainage. Elevated ICP can therefore result from any mechanism that increases the volume of any of the three components. Alternatively, ICP can also increase by the addition of a fourth component, such as a mass, intracranial hemorrhage or cerebral edema that expands beyond the ability of the system to compensate by decreasing the volume of another. 

Some changes in mean ICP are expected under regular physiologic conditions, including changes in posture, brain activity, cardiovascular function, respiratory function and adrenergic tone [7,8,9,10]. Since some variability in ICP is expected, clinical use of ICP monitoring uses a time-averaged ICP to establish baseline, with overnight measurement over at least 30 minutes considered to be the ‘gold standard’ in non-comatose patients [11]. Similarly, alterations in ICP reach clinical significance when they are sustained longer than at least 5 min. Physiologic boundaries of mean ICP are 7–15 mm Hg in supine adults, 3–7 mm Hg in children and 1.5–6 mm Hg in infants, though mean ICP in pediatric populations may vary depending on age and are not as well established [12]. The maintenance of ICP within its physiologic boundaries is of critical importance to prevent brain injury [13,14,15]. Elevated ICP-related injury occurs primarily via one of two mechanisms: (1) cerebral ischemia and (2) brain herniation. Cerebral blood flow (CBF) is tightly linked to cerebral perfusion pressure (CPP), which is governed by both mean arterial pressure (MAP) and ICP through the following relationship, CPP = MAP-ICP. Accordingly, as ICP increases, MAP is increased, primarily through a rise in cardiac output, in order to maintain a steady CPP. In the presence of elevated ICP beyond the ability for compensation through elevation of MAP, CPP will be compromised and cerebral ischemia may follow. While under the Monro–Kellie hypothesis, the intracranial space is a constant, enclosed space, the brain and intracranial CSF continue, of course, through the foramen magnum at the base of the skull to become the brainstem, spinal column, and the CSF-filled spinal canal. When ICP is sufficiently elevated, the pressure differential between the intracranial cavity and the spinal canal can cause the downward motion of brain tissue (i.e., herniation), which can compress vital brainstem structures, and subsequently lead to severe neurological outcomes including death [15,16,17].

**Figure 2:**
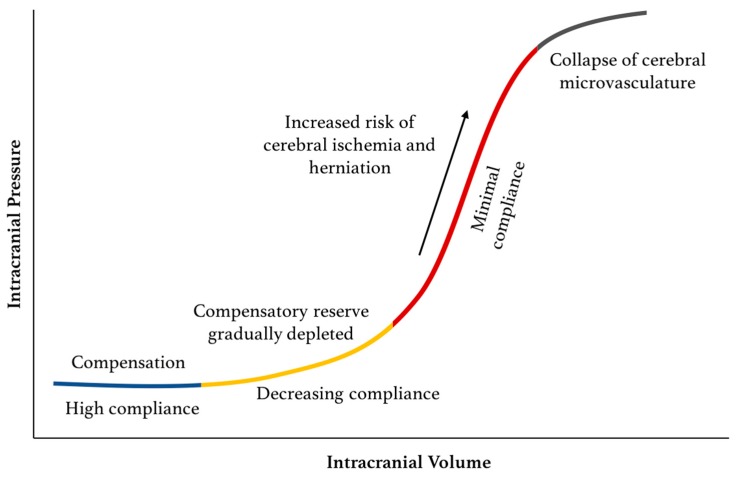
Pressure–volume curve for ICP. The pressure–volume curve has four ‘zones’: (1) baseline intracranial volume with good compensatory reserve and high compliance (blue); (2) gradual depletion of compensatory reserve as intracranial volume increases (yellow); (3) poor compensatory reserve and increased risk of cerebral ischemia and herniation (red); and (4) critically high ICP causing collapse of cerebral microvasculature and disturbed cerebrovascular reactivity (grey).

**Figure 3:**
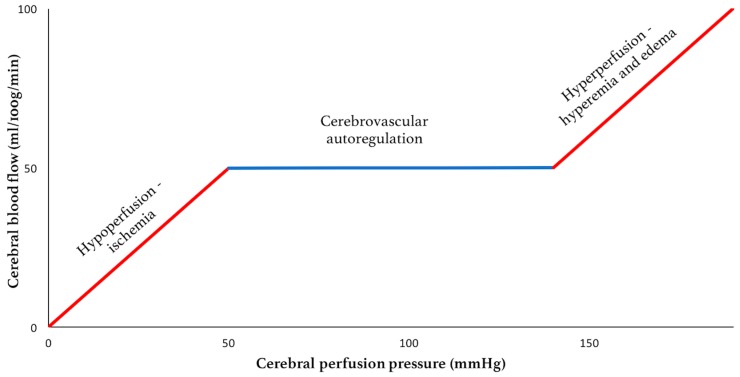
Cerebral autoregulation capacity.

As some variability in ICP is expected even under physiologic conditions, there are intrinsic compensatory mechanisms to maintain a stable mean ICP. Foremost among these is that ability to modify the brain venous blood pool. Additionally, there is an ability, albeit limited, of some CSF to expand further out of the intracranial space and into the spinal canal [18]. This compensatory reserve is finite and is dependent on the compliance of the system. When the reserve is depleted, small elevations in volume will lead to potentially dangerous sustained elevations in ICP (Figure 2). Alongside these mechanisms to attenuate changes in ICP, cerebrovascular autoregulation functions to maintain the necessary CPP in the face of ICP changes by way of altering cerebral arteriolar resistance. Autoregulation, however, is only effective between a CPP of 50–150 mmHg, below and above which hypoperfusion and cerebral edema may ensue, respectively (Figure 3). In addition, autoregulatory capacity is also dependent on arterial pressure of carbon dioxide (PaCO_2_). Hypercapnia causes dilation of the cerebral vessels leading to an increase in CBF and a risk of hyperperfusion. Conversely, hypocapnia causes vasoconstriction, which may result in ischemia [19,20]. 

The early characterization of the components of ICP and its importance for clinical outcomes led to a desire to measure ICP for the purpose of guiding clinical management. The earliest surgical approach to reduce ICP was through the use of the external ventricular drain (EVD), which was used to drain CSF in pediatric patients with congenital hydrocephalus [21]. It was only around the turn of the 20th century that EVDs could be placed safely, and with aseptic technique to avoid iatrogenic intracranial infections [22]. Soon thereafter, the first instance of ICP monitoring using an EVD-based manometric system was described by Adson and Lillie in their landmark 1927 paper [23]. Since that time, the indication for ICP monitoring has expanded, and, currently, the most common neurological and neurosurgical pathologies that require ICP monitoring include traumatic brain injury (TBI), subarachnoid hemorrhage (SAH), and hydrocephalus.

## 2. Invasive ICP Monitoring

Methods for ICP monitoring can be divided into invasive and non-invasive approaches. Invasive methods include fluid-based systems and implantable micro-transducers. Non-invasive methods, most of which offer indirect measurement of ICP, will be discussed later in this text. Of the invasive methods, ICP monitoring using an EVD is considered as the gold standard, not only for its accuracy but also because it additionally serves a therapeutic purpose by allowing CSF drainage [11,24]. EVDs allow for fluid-based monitoring as the pressure in the catheter equilibrates with the intraventricular pressure. This pressure transmits into an external saline-filled tube through a stain-gauge transducer from which the pressure measurement is made. The insertion of an EVD may be difficult in patients with inherently small ventricles size or those with ventricular compression attributable to advanced brain swelling [11,24]. Additionally, there is a 5–7% risk of hemorrhage during insertion [25,26]. EVDs are not suitable for long-term ICP monitoring since risk of intracranial infection starts to increase with an estimated overall risk of 5% after five days [11]. Another fluid-based system is the subarachnoid screw, which is inserted through a hole drilled in the skull whose tip projects through the dura into the subarachnoid space [27,28]. These devices, however, cannot drain CSF and have a considerable risk of local wound infection [29].

**Figure 4:**
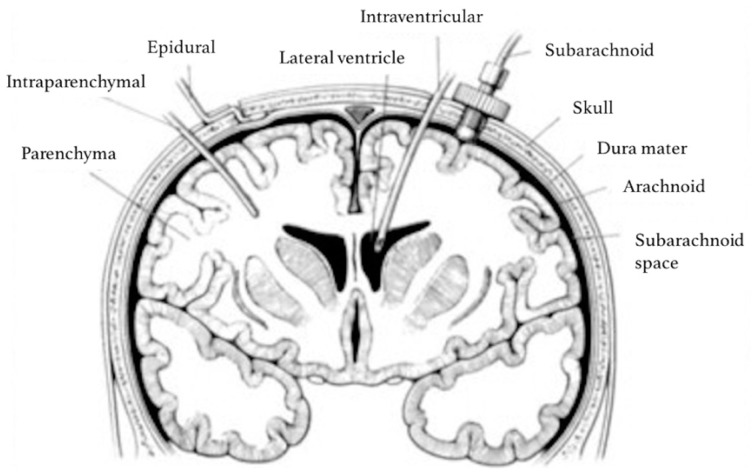
Sites for invasive ICP monitoring. These sites represent actual and potential spaces in the intracranial cavity in which ICP can be measured. Intraventricular monitoring with EVDs is the most commonly accessed site in clinical practice, followed by intraparenchymal probes. Reproduced with permission [32].

ICP can also be measured using implantable microtransducers such as strain gauge devices, pneumatic sensors and fiber-optic sensors (technical review by Zhang et al. [30]). In strain gauge devices, ICP changes cause the diaphragm to bend, leading to changes in the electrical resistance that are used to calculate ICP [31]. Pneumatic sensors have a balloon in the distal end of the probe, where pressure exerted on the balloon is equal to the pressure of the surrounding tissue (i.e., ICP). Pneumatic sensors have also been used to measure intracranial compliance [31]. In fiber-optic sensors, changes in ICP move a displaceable mirror at the tip of the sensor, altering the intensity of the light reflected back along the fiber optic cable [27,31]. Most micro-transducers probes tips are placed intraparenchymally, but these can also be placed in the intraventricular, subarachnoid, subdural or epidural compartment (Figure 4). Advantages of implantable microtransducers are lower infection rates and risks of hemorrhage compared to EVDs [11]. However, these are more expensive and, with the exception of pneumatic sensors, generally cannot be recalibrated once in situ, which can affect the precision of ICP measurements [24,29,31]. Generally, micro-transducers are used in situations where EVD placement is not successful or when clinicians judge that CSF drainage is not likely to be necessary.

While the management of ICP is of clear clinical benefit, there is no consensus in the literature about whether ICP monitoring provides clinical benefit as compared to management based solely on the patient’s neurological exam, imaging findings and clinicians’ acumen. While some studies demonstrate that ICP monitoring is associated with improved survival [33,34,35,36,37,38,39,40,41], others suggest that ICP monitoring is not only not beneficial, but may, in fact, lead to worse outcomes. Specifically, in some studies, ICP monitoring was associated with a significant increase in mortality, longer hospital length of stay, complication rate and increased utilization of hospital resources, compared to patients managed without ICP monitoring [42,43,44,45,46,47]. The only randomized controlled trial of ICP monitoring in patients with traumatic brain injury (TBI) was conducted by Chesnut et al. in 2012 [48]. The authors compared outcomes between patients whose treatment was guided by imaging and clinical exam alone, and those who additionally received invasive ICP monitoring. The overall 6-month mortality rate was approximately 40%, with no survival benefit seen in patients who received ICP monitoring compared to those in whom treatment was guided only by neurologic examination and serial CT imaging. Taking these in sum, there remains room for improvement of the clinical utility of ICP monitoring in critically ill patients.

## 3. Approaches to Improving Utility of Invasive ICP Monitoring

### 3.1. Cerebral Compliance and ICP Waveform Analysis 

Current guidelines for ICP management primarily use mean ICP as the main metric to guide therapy [16,49]. Given the shape of the intracranial pressure–volume curve (Figure 2), a reliance solely on mean ICP for clinical management fates the practice to be reactive rather than proactive [50]. Many have argued that this is the reason why ICP monitoring has not provided more clinical benefit than initially hoped [18]. Accordingly, research in this field has shifted its attention to determining *where* along the pressure–volume curve the patient is at any given time, and, specifically, to measure intracranial compliance and track the depletion of the system’s compensatory reserve [18,51,52]. 

**Figure 5:**
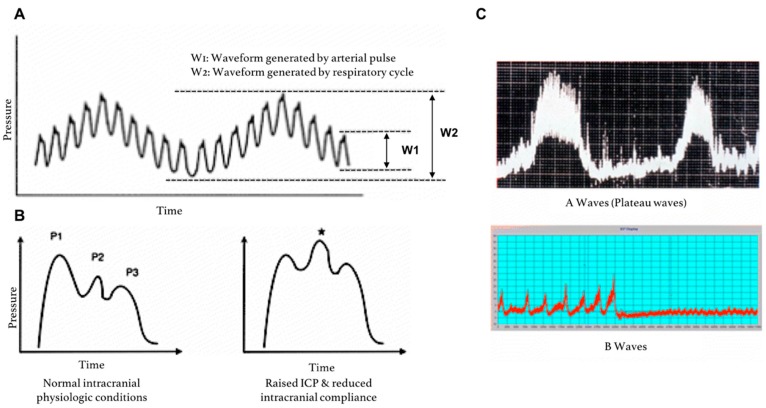
ICP pressure waves. (**A**) ICP fluctuations in response to the respiratory cycle (W2) and the arterial cycle (W1); (**B**) close-up of ICP waveform due to the systemic arterial cycle. Components are P1 (Percussion wave = representative of arterial pulsation), P2 (Tidal wave = a proxy for intracranial compliance) and P3 (Dicrotic wave = pressure transmission of aortic valve closure). A raised P2 wave is an indicator of raised ICP and reduced intracranial compliance (*); (**C**) Lundberg A (plateau) and B waves; adapted from Hall et al. [55].

Mean ICP is a time-average of the ICP waveform (Figure 5A). ICP waveform can be visualized in the neuro-intensive care setting using software like Odin Monitoring System, ICM^+^, Sensometrics and the ICU Pilot system [53]. The ICP waveform consists of three components—(1) respiratory waveforms (0.1–0.3 Hz) associated with the respiratory cycle (W2), (2) pulse pressure waveforms (frequency equal to heart rate), and (3) slow vasogenic waveforms (e.g., ‘Lundberg A and B waves’) [54]. The pulse pressure waveform is itself subdivided into three waves (Figure 5B). Elevated ICP not only increases mean ICP, but also affects the characteristics of the ICP waveform. Specifically, elevated ICP is associated with a relative increase in the P2 component of the arterial wave, which is thought to represent decreased intracranial compliance. Additionally, the presence of Lundberg A waves, which are sustained sharp increases in mean ICP lasting 5–20 min, may also signify diminishing compliance (Figure 5C) [55]. Lundberg B waves, which are clustered cyclic elevations in ICP occurring at a rate of 0.33–3 cycles per minutes with overall cluster duration of 5–30 min [56,57], are non-specific indicators of diminished compliance as they can also be present in patients with normal ICP [55].

The pulse amplitude (AMP) of the arterial cycle, which can be isolated using spectral analysis of the waveform, has been shown to be a useful index, where higher AMP is associated with lower compliance [11,55]. The RAP coefficient is a correlation coefficient (R) between the AMP amplitude (A) and mean ICP (P), which has also been proposed as a measure of compensatory reserve [11,18,55]. The RAP has a value of 0 in the linear part of the “pressure–volume curve” at low ICP, which indicates a good compensatory reserve (e.g., brain compliance). The RAP has a value of +1 in the ascending exponential part at moderately increased ICP, which indicates a low compensatory reserve. When ICP increases even further, AMP will decrease due to disturbed CBF and the collapse of the cerebral microvasculature, causing RAP to become negative [18,55,58]. While there is still no clear consensus in the literature regarding whether AMP or RAP are more accurate in assessing cerebral compensatory reserve [55,59], a recent study demonstrated that RAP-weighted ICP shows significant association with outcomes in TBI patients [58].

While a qualitative evaluation of ICP waveform is a part of current clinical practice, there are no widely-used computational tools to quantitatively analyze these continuous data streams outside the realm of research. A number of machine learning and deep learning algorithms have been proposed and trialed as potential approaches to this data [60,61,62,63,64,65,66]. A recent study demonstrated about 92% accuracy in detecting elevated ICP using only waveform characteristics with a deep learning algorithm [67]. Similarly, a multi-center study conducted by the BrainIT group demonstrated the ability of a machine-learning based model to predict ICP elevations 30 minutes prior using a combination of ICP and MAP data from the preceding four hours, supporting the prospects of this methodologic approach to continuous monitoring data [68,69,70]. 

### 3.2. Autoregulation

As mentioned earlier, cerebrovascular autoregulation is a key intrinsic mechanism designed to maintain a constant CPP in the face of changing ICP [7,71]. It has been proposed that a focus on cerebrovascular autoregulation and CPP monitoring, as an adjunct to ICP monitoring, may be beneficial for clinical care [72,73,74,75]. While there are several indices of cerebrovascular autoregulation (reviewed by Donnelly et al. [7]), the most commonly used is the pressure reactivity index (PRx), which is the time-averaged correlation coefficient between ICP and arterial blood pressure (MAP) [74]. A positive PRx indicates an impaired autoregulatory capacity of the brain, whereas a negative PRx reflects a normal autoregulatory capacity [11,74]. The use of autoregulation-weighted ICP and CPP, which calculate patient-specific ICP and optimal CPP thresholds based on PRx, have been proposed [72,74]. Patient-specific ICP thresholds were shown to be stronger predictors of mortality than the fixed ICP thresholds ranging from 20–25 mm Hg [72]. Similarly, a recent review suggested that the proximity of measured CPP and the calculated weighted optimal CPP were associated with improved outcomes, but that more rigorous studies are still needed to verify this trend [74].

### 3.3. Brain Oxygenation 

The primary danger of elevated ICP alongside impaired cerebrovascular autoregulation is the development of brain ischemia and subsequent hypoxia. Some studies have suggested that hypoxia can also occur in TBI patients due to disrupted diffusion rather than ICP-attributable perfusion defects [76,77,78]. Accordingly, it has been proposed that monitoring brain tissue oxygenation (PbO_2_) will provide a more proximate estimation of tissue health and might therefore be more directly linked with patient outcomes than mean ICP. This hypothesis was tested in the recently concluded randomized controlled BOOST-II trial [78]. Severe TBI patients were randomized into two groups: ICP-only group or ICP + PbO_2_-guided management group. Both the ICP probe and the PbO_2_ probes were placed intraparenchymally. The study showed that a multimodal approach using PbO_2_ monitoring alongside ICP reduced brain tissue hypoxia led to decreased mortality and more favorable outcomes compared to ICP monitoring alone [78]. The impact of this multimodal approach on neurologic outcome will be further assessed in the upcoming BOOST-III trial.

Near-infrared spectroscopy (NIRS) is a non-invasive technology that has been under development for assessment of cerebral oxygenation and ICP [79]. NIRS sensors emit NIR light onto the surface of the head and detect the reflected light. Changes in the underlying tissue characteristics affect light absorption and diffusion, and subsequent spectral analysis can be used to garner information about tissue state [79]. The technology has been successful in monitoring oxygenation in cardiac and vascular procedures, as well as in pediatric populations, as skull characteristics are particularly amenable. However, there have been barriers to the implementation of this technique in TBI patients due to the effect of scalp and skull injury, as well as pathological changes in baseline saturation that have thus far made the technology less reliable in these settings [79,80,81].

## 4. Non-Invasive ICP Monitoring

Invasive methods for ICP monitoring are currently the most accurate way to measure ICP [31]. Additionally, other than providing diagnostic information, EVDs also serve a therapeutic benefit through the drainage of CSF; thus, despite the risks associated with the placement of invasive ICP monitors, these remain necessary in the majority of critically ill patients [11,24,29]. There are, however, specific clinical circumstances and populations where non-invasive methods to assess ICP would be desirable. For one, non-invasive monitoring methods can be used to screen patients for elevated ICP in situations where invasive interventions cannot be promptly accessed, such as in the field or where there are no neurosurgeons. Additionally, non-invasive screening can be done in patients in whom there is relatively low suspicion of elevated ICP, but the possibility needs to be ruled out. This may decrease the placement of invasive monitors in patients who, in retrospect, did not need them. 

### 4.1. Transcranial Doppler (TCD)

In the neuro-critical setting, transcranial Doppler (TCD) is most commonly used as a tool to monitor changes in cerebral blood flow (CBF) in the setting of subarachnoid hemorrhage-associated vasospasm. A number of models using TCD-derived data have shown correlation with invasively-measured ICP; these models have used measurements of flow velocity (FV) in the middle cerebral artery, arterial blood pressure and pulsatility index (PI) [82,83,84,85,86,87,88]. A recent prospective, head-to-head study found that using a model that combines these approaches is superior to either individually in estimating ICP [85]. The combined model-derived ICP estimate correlated with invasive ICP measurements (*R* = 0.47; *p* < 0.05) and performed with an area under the curve of 0.73 (*p* < 0.05). While computational modeling continues to make TCD-based ICP estimates more accurate, the technique has some inherent limitations to its widespread integration into clinical care. Like most ultrasonographic techniques, TCD is prone to intra- and inter-observer variability. Additionally, it provides a one-time measurement, and while it has potential as a screening tool, it will not be sufficient in patients requiring continuous monitoring. Lastly, in 10–15% of patients, skull characteristics limit transmission of ultrasound waves, making TCD difficult to interpret [31]. TCD-based assessment of CBF and autoregulation has been more successful than TCD-based ICP estimations, and so this technology may be incorporated into clinical practice sooner as a neuro-monitoring adjunct rather than an ICP sensor [89].

### 4.2. Optic Nerve Sheath Diameter (ONSD)

When the optic nerve exits the intracranial space into the orbit, it is still surrounded by the dural sheath. As such, the subarachnoid space surrounding the nerve is contiguous with the intracranial subarachnoid space [90]. Elevation in ICP can transmit through the CSF in the subarachnoid space, leading dilatation of the optic nerve sheath, which can be detected using transocular ultrasonography [31]. Several studies have demonstrated a correlation between invasively measured ICP and ultrasonographic ONSD measurements, with overall sensitivity and specificity of 0.90 and 0.85, respectively [91,92,93,94,95,96]. A recent prospective study demonstrated similar sensitivity and specificity and suggested a diameter of 5.6 mm as the optimal cut-off for diagnosing elevated ICP [94]. While intra- and inter-observer variability seems to be lower than that for TCD [91,94,97], this technology cannot be used in patients with face trauma or with medical conditions that may otherwise affect ONSD (e.g., Grave’s disease, sarcoidosis) [31]. Additionally, there is some suggestion that the specificity of ONSD wanes when there are acute fluctuations in ICP [98]. Nevertheless, ONSD measurements may become useful as a screening test for ICP in settings where invasive monitoring is not promptly available.

Optical coherence tomography (OCT) is another ophthalmologic approach that has been trialed for ICP measurement. It too depends on the transmission of ICP through the optic nerve sheath and has shown particular promise in assessing ICP in children [99].

### 4.3. Imaging-Based Methods

There are a variety of gross anatomic changes associated with elevated ICP that can be detected using computed tomography (CT) and magnetic resonance imaging (MRI). For instance, the presence of a mass occupying lesion can cause compression of the ventricles and midline shift. Similarly, enlarged ventricles can be indicative of hydrocephalus, and cerebral edema can cause a loss of differentiation of grey and white matter junctions [100]. CTs and MRIs are routinely used for diagnostic purposes and can provide qualitative information about ICP. In a small pilot study, an MRI-based technique to estimate ICP by assessing net transcranial blood and CSF flow was able to differentiate patients with normal or elevated ICP [101]. Another small study in TBI patients demonstrated an ability to differentiate normal and elevated ICP in a subset of patients using a CT-determined ratio of CSF volume to the total intracranial volume with a predictive accuracy of 67% [102]. While imaging continues to be used qualitatively, at present, these methods are not independently reliable enough as screening tools for elevated ICP [31,103].

### 4.4. Telemetric Sensors

Long-term ICP monitoring is needed to detect shunt malfunction in patients with implanted ventricular-peritoneal (VP) shunts, and to assess ICP in patients with chronic intracranial hypertension disorders. Risks of infection precludes these patient populations from having long-term transcranial monitoring (e.g., EVD, bolts), and, therefore, implanted telemetric sensors have been investigated as a possible solution [104,105,106,107,108]. Several different sensors have been trialed over the years, and the two commercially available sensors are both strain-gauge micro-transducers composed of a housing unit that sits subcutaneously and an element that extends intracranially through a small burr hole in the skull. In the Neurovent P-tel (Raumedic, Helmbrechts, Germany), the sensor is an intraparenchymal micro-transducer [104], while the Sensor Reservoir (Miethke , Potsdam, Germany) is a CSF reservoir-integrated unit connected to an intraventricular catheter [109]. In both systems, changes in circuit resistance generated by changes in ICP are recorded on a microchip, and the information can subsequently be read by the clinician using an external device. Both technologies have shown promise for the purpose of using sensor-derived ICP information to guide valve adjustments in chronically-shunted patients [104,109]. While there were early attempts to use shunt-integrated ICP sensors based on CSF flow [110], there remains a long-standing need for this technology that would obviate the need for additional implanted devices. 

## 5. Conclusions

The management of elevated ICP has long been known to affect clinical outcomes in patients suffering from a range of neurological conditions. There remains room for improvement in ICP monitoring systems that would provide more actionable information and improved clinical benefit. For invasive ICP monitoring approaches, the directions for improvement primarily lie with a multimodal approach, integrating metrics such as brain autoregulation and oxygenation. In this sphere, there is therefore a hardware need for integrated sensors, as well as a computational need for algorithms to process and analyze multiple continuous streams of neuro-monitoring data. Additionally, continued development of non-invasive ICP sensors has the potential of decreasing the need for invasive interventions in a range of patients.
